# Philadelphia chromosome-positive B-cell acute lymphoblastic leukemia with e13a3 fusion transcripts in a patient with pre-existing essential thrombocythemia: a case report and literature review

**DOI:** 10.3389/fonc.2025.1573893

**Published:** 2025-05-01

**Authors:** Ying Wu, Dan Cao

**Affiliations:** ^1^ Department of Hematology, Huzhou Central Hospital, Fifth School of Clinical Medicine of Zhejiang Chinese Medical University, Huzhou, Zhejiang, China; ^2^ Department of Hematology, Huzhou Central Hospital, Affiliated Central Hospital of Huzhou University, Huzhou, Zhejiang, China

**Keywords:** essential thrombocythemia, Philadelphia chromosome positive, acute lymphoblastic leukemia, e13a3 fusion, olverembatinib

## Abstract

Essential thrombocythemia (ET), a *BCR-ABL1*-negative myeloproliferative neoplasm (MPN), is characterized by persistent thrombocytosis and excessive megakaryocytic proliferation in the bone marrow. During the course of the disease, 4% of patients progress to acute leukemia, the majority of which have acute myeloid leukemia (AML), and are confirmed to be transformed from ET. Transformation to acute lymphoblastic leukemia (ALL) is exceedingly rare, with limited evidence clarifying its clonal relationship to antecedent ET. We report a case of a 66-year-old man with a history of ET, lacking mutations in Janus kinase 2 (*JAK2*), Calreticulin (*CALR*), or myeloproliferative leukemia virus oncogene (*MPL*), who subsequently developed Philadelphia chromosome (Ph)-positive B-cell ALL (B-ALL), harboring a rare e13a3 fusion transcript. Following four cycles of induction therapy with olverembatinib, vincristine, and prednisone, the patient achieved complete hematologic and molecular remission. A chemotherapy-free consolidation therapy with olverembatinib and blinatumomab maintained sustained complete molecular remission at follow-up. To our knowledge, this represents the first case of Ph-positive B-ALL with e13a3 transcripts arising in a patient with preexisting ET, providing critical therapeutic insights for managing similar cases.

## Introduction

Essential thrombocythemia (ET), a clonal disorder, arises from clonal expansion of hematopoietic stem cells, leading to sustained thrombocytosis and pathological megakaryocytic hyperplasia in the bone marrow (BM). Patients with ET face dual risks of thrombotic and hemorrhagic complications (1). While the median survival of ET is approximately 20 years, extending to 32.7 years for patients under 60 years, it remains reduced compared to age- and sex-matched healthy controls (2, 3). Compared to other myeloproliferative neoplasms (MPNs), leukemic transformation during the course of ET is rare, with a cumulative incidence of approximately 4%–5% over 20 years (4). However, it carries a dismal prognosis. Patients typically exhibit resistance to conventional chemotherapy, and allogeneic hematopoietic stem cell transplantation (allo-HSCT) remains the only curative option (5). Most patients with post-ET leukemia have acute myeloid leukemia (AML), with transformation to acute lymphoblastic leukemia (ALL) being exceptionally rare (5). The mechanism is not well understood and may include treatment-related factors, such as the use of hydroxyurea and alkylating agents, and high-risk gene mutations, including *SRSF2*, *SF3B1*, *U2AF1*, and *TP53* (5, 6). Here, we report the first documented case of Philadelphia (Ph)-positive B-cell ALL (B-ALL) with e13a3 *BCR::ABL1* fusion transcripts that followed triple-negative ET. Importantly, this patient achieved sustained complete molecular remission (CMR) through a chemotherapy-sparing regimen combining olverembatinib and blinatumomab, challenging the historical reliance on intensive chemotherapy or allo-HSCT in this high-risk population.

## Case report

**Figure 1:**
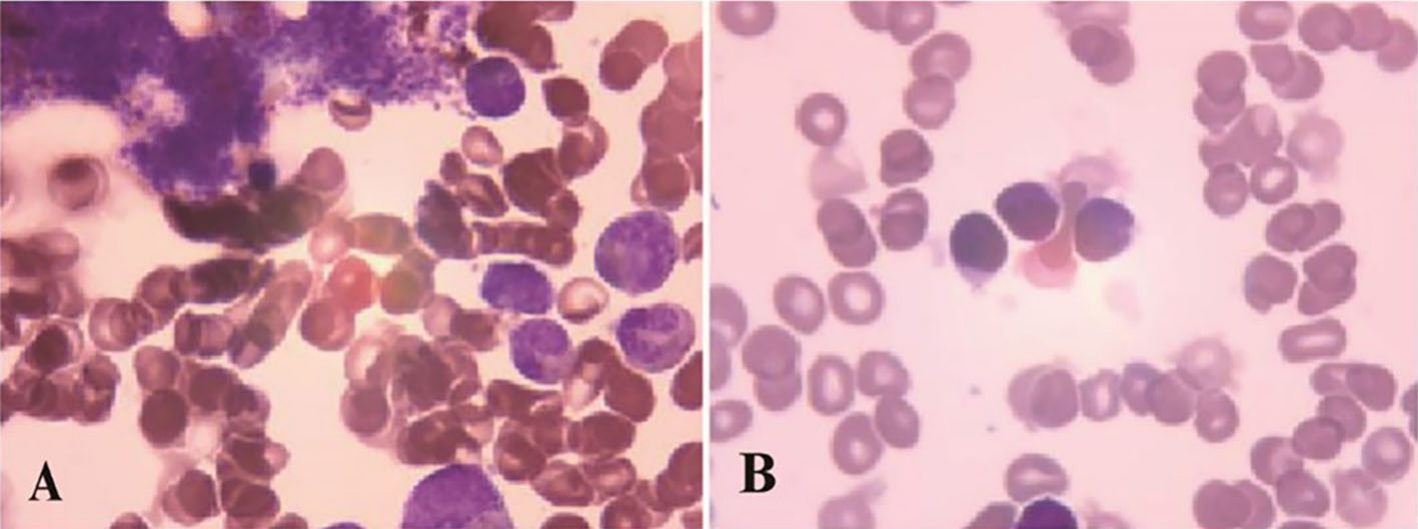
Bone marrow examination results: **(A)** 2022-1-11, morphological feature of ET **(B)** 2024-2-21, morphological feature of ALL.

**Figure 2:**
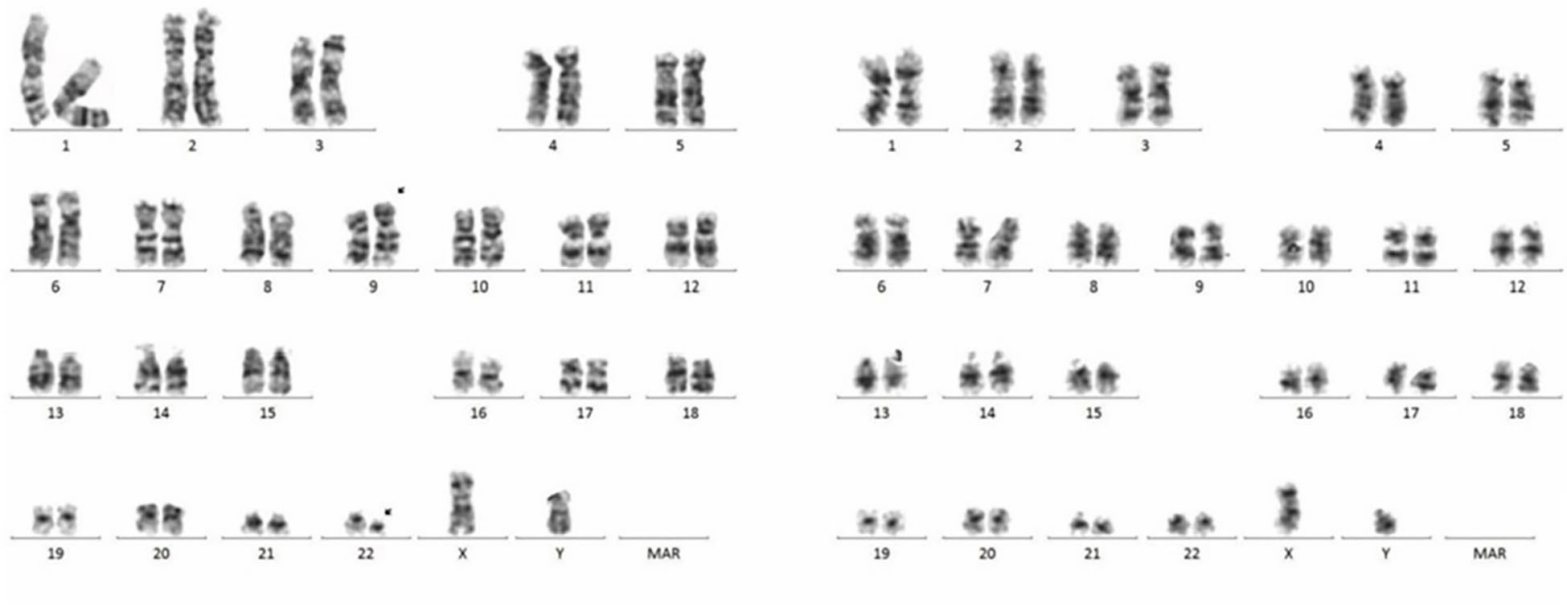
Chromosome analysis: 46,XY,t(9;22)(q34;q11.2)[19]/46,XY[1].

**Figure 3:**
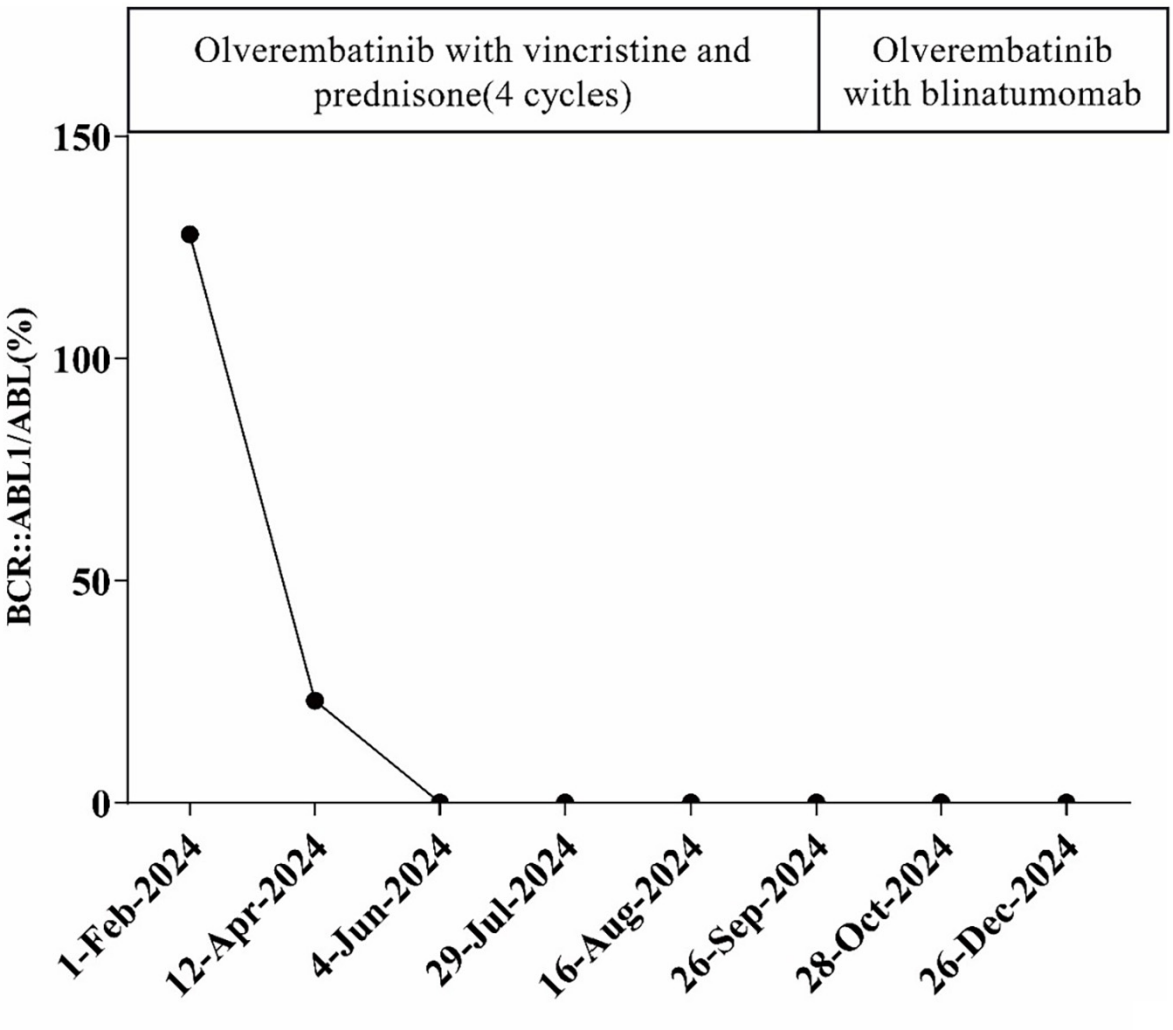
Treatment regimen and the percentage of *BR::ABL1* transcripts during the course of the disease.

A 66-year-old male was incidentally found to have an elevated platelet count during a routine medical examination in 2022. The patient denied any clinical symptoms such as headaches, dizziness, chest pain, or paresthesia in the extremities. A complete blood count (CBC) showed a white blood cell count (WBC) of 6.2 × 10^9^/L, hemoglobin of 13.5 g/dL, and platelets of 674 × 10^9^/L. Physical examination was normal. The patient’s medical history was unremarkable for chronic illnesses, cardiovascular risk factors (hypertension, diabetes, or dyslipidemia), or familial predisposition to hematologic malignancies or inherited blood disorders. He denied tobacco use, alcohol consumption, or chronic exposure to medications or toxins. The BM aspiration showed an increased number of megakaryocytes with focal clustering (**Figure 1A**), while a biopsy showed 75% cellularity with grade 0 reticulin fibrosis. Megakaryocytes were large with hyperlobulated nuclei and abundant cytoplasm. No significant hyperplasia or left shift was observed in the granulocytic and erythroid series. Cytogenetic analysis revealed a normal diploid male karyotype. Molecular studies were negative for *JAK2 V617F*, *CALR*, and *MPL* mutations, as well as *BCR::ABL1* fusion by reverse transcriptase-polymerase chain reaction (RT-PCR). ET was confirmed per WHO 2022 criteria: sustained thrombocytosis (≥450 × 10^9^/L); the exclusion of other myeloid neoplasms, including prefibrotic primary myelofibrosis (PMF), polycythemia vera, chronic myeloid leukemia, and myelodysplastic syndromes; megakaryocytic hyperplasia without dysplasia; and exclusion of reactive and secondary causes. The patient subsequently received treatment with hydroxyurea and aspirin, during which platelet counts fluctuated between 200–450 × 10^9^/L. In February 2024, the patient was admitted to the hospital due to diarrhea, fatigue, and fever for 5 days. A CBC at the local hospital showed a WBC of 67.7 × 10^9^/L, hemoglobin of 10.7 g/dL, and platelets of 46 × 10^9^/L. Peripheral blood smear revealed 9% abnormal cells. The patient was subsequently admitted to our hospital. A BM examination showed hypercellularity with increased numbers of blasts consistent with B-ALL (**Figure 1B**). Flow cytometry identified B-lymphoblasts accounting for approximately 91.489% of the leukocytes, expressing CD19, CD34, CD123, CD20, CD22, CD10, nTdT, cyCD79a, CD81, and CD9, but lacking CD117, CD7, CD13, CD33, CD38, CD56, CD5, sIgM, CD200, CDC66c, and cIgM. Karyotype analysis showed 46, XY, t(9;22)(q34; q11.2)[19]/46,XY[1] (**Figure 2**).Molecular studies confirmed the e13a3 *BCR::ABL1* transcript. A 365-panel next-generation sequencing (NGS) revealed two mutations: *SETD2* c.5126_5127insCC [variant allele frequency (VAF) 89.4%] and *BTK* c.1408A > G (VAF 1.5%). Thus, a diagnosis of Ph+ B-ALL was confirmed. At the time of the ET diagnosis (2022), the patient’s medical history was unremarkable for chronic illnesses or cardiovascular risk factors. However, during hospitalization for B-ALL progression in 2024, the patient developed acute chest pain. Comprehensive cardiac evaluation, including an electrocardiogram (ECG), cardiac biomarkers, echocardiography, and coronary computed tomography angiography (CTA), revealed ST-segment depression, troponin elevation, and microvascular dysfunction. A cardiology consultation confirmed the diagnosis of unstable angina. The patient was managed with dual antiplatelet therapy (aspirin and clopidogrel), atorvastatin, and isosorbide mononitrate. Given his age, unstable angina, and preference for minimal treatment disruption, we opted for a chemotherapy-sparing regimen combining olverembatinib (40 mg every other day) with vincristine and dexamethasone. One month later, a repeat bone marrow examination indicated morphological remission, and minimal residual disease (MRD) of 0.232% (1293/557378) by flow cytometry. Karyotype analysis showed 46,XY,t(9;22)(q34;q11.2)[12]/46,XY[8]. *BCR::ABL1* transcript levels decreased to 22.9%. The patient declined allo-HSCT. Following three consolidation cycles with olverembatinib and VP (vincristine and prednisone), sustained CMR was achieved. The patient is currently receiving maintenance therapy with olverembatinib and blinatumomab (a bispecific anti-CD3/anti-CD19 T cell engager), and the disease remains in molecular remission. The treatment regimens and percentage of e13a3 fusion transcripts are illustrated in **Figure 3**. The patient will undergo monthly *BCR::ABL1* PCR monitoring for 12 months, transitioning to quarterly assessments if the remission is sustained. A bone marrow evaluation (morphology, flow cytometry, and cytogenetics) will be carried out every 3 months for 2 years.

## Discussion

Leukemic transformation in ET is uncommon, with an estimated 10-year cumulative incidence of <1% (7). Recognized risk factors include *JAK2* mutations, chromosomal abnormalities, and extreme thrombocytosis (platelet count ≥1000 × 10^9^/L) (8, 9). Most patients transform to AML and transformation to ALL is rare, with only case reports available. O’Hea et al. reported a case of B-ALL following ET in 1986 (10). In 1996, Berkahn et al. reported a case of T-ALL transformation from ET (11). Nagai et al. reported a case of Ph+ B-ALL that developed after a long duration of ET, and mutational analysis of *JAK2* showed that the B-ALL clone did not originate from the ET clone with the *JAK2-V617F* mutation (12). A case of a patient with Ph+ALL, developed from MPL-mutated ET, was reported by Ma et al. in 2021. Molecular studies also indicated that the clone did not originate from the ET clone carrying the *MPL* p.(*W515L*) variant (13). In 2017, Hilal et al. reported a case of B-ALL with der(1)t(1;19)(p13;p13.1) arising in the setting of *CALR* exon 9-mutated ET (14). Medawar et al. reported a case with a history of *JAK2/CALR*-negative ET in which the patient subsequently developed T-ALL (15). Thus, including the case reported here, there have been seven reported cases of acute lymphoblastic leukemia transformation following ET (**Table 1**). The average age at which the patients developed ALL was 61 years (range: 49–87 years), with five cases transforming to B-ALL, two cases to Ph+ ALL, and two cases to T-ALL. The median time to progression was 11.4 years (range: 2 months–26 years). At the time of ET diagnosis, four patients underwent mutation testing (JAK2/CALR/MPL), revealing two triple-negative cases, one with a *JAK2* mutation, and one with a *CALR* mutation. At the time of transformation, NGS was performed in three patients, identifying mutations in *SETD2* (c.5126_5127insCC), *BTK* (splice site c.1408A > G), *MPL* p.(W515L), *TET2* (R1261C), *NOTCH1* (L1678P), and *PHF6* (splice site c.374 + 1G > A).

**Table 1:** Characteristics of cases of ALL following ET.

	Our case	A M O'HEA et al	Berkahn et al	Nagai Y et al	Jiale Ma et al	Hilal T et al	Georgio Medawar et al
age(year)	66	61	87	67	71	69	49
gender	male	female	female	female	female	female	male
pathogenic muation	negative	N/A	N/A	JAK2-V617F	N/A	CALR exon 9	negative
treatment of ET	hydroxyurea and aspirin(2 years)	5 mCi 32p	Hydroxyurea	Hydroxyurea (8years) and anti thrombotic agent (more than ten years)	hydroxyurea and antiplatelet therapy for 9 years	hydroxyurea, followed by anagrelide, and ruxolitinib for 1 month	Hydroxyurea
time of ET to ALL	2 years	16 years	2 months	16 years	10 years	26years	10 years
ALL type	Ph+ ALL	B-ALL	T-ALL	Ph+ALL	B-ALL	B-ALL	T–ALL
Karyotype of leukemic transformation	46,XY,t(9;22)(q34;q11.2)[19]/46,XY[1]	44 XX with absent chromosomes 7, 21, and 14q+, 6p-.	46, XX	Ph chromosome and monosomy 7	46,XX,t(9;22)(q34;q11)[8]/ 46,XX,[2]	47,XX,t(1;19)(p13;p13.1), +der(1)t(1;19)[3]/46,XX[17])	46,XY,− 10,− 14,+2mar[6]/49~ 56, XY,+5,+15,+16,+19,+20,+20[cp2]/46, XY[12]
NGS	*SETD2*(c.5126_5127insCC, VAF 89.4%, read depth 2060) and *BTK* (splice site c.1408A > G,VAF 1.5%, read depth 1150).	N/A	N/A	N/A	*MPL* p.(W515L,VAF 2.59% ) and *TET2* (R1261C, VAF 49.91%)	N/A	*NOTCH1* (L1678P), *PHF6* (splice site c.374+1G >A)
treatment of ALL	olverembatinib with VP(3 cycles) followed by olverembatinib and blinatumomab	supportive care	supportive care	dasatinib and prednisolone	flumatinib and prednisolone,followed by flumatinib maintenance	supportive care	Hyper-CVAD (4 cycles), followed by HSCT
outcome	alive with CMR	death after 5 months	rapid deterioration and death	relapsed with the T315I mutation nine months later	relapsed and died after 6 months	death after 3 months	remission at 4th month

N/A, Not Available.

The clonal origins of post-ET ALL remain controversial. In our case, the absence of shared driver mutations (*JAK2/CALR/MPL* in ET vs. *SETD2/IKZF1* in ALL) supports clonal independence, consistent with prior reports of *MPL*- or *JAK2*-mutated ET progressing to Ph+ B-ALL (12, 13, 15). Notably, similar findings were observed in *MPL*-mutated ET transforming to Ph+ ALL, where the low VAF of MPL in leukemic blasts (2.59%) contrasted with the dominant *BCR::ABL1* clone, further indicating divergent clonal evolution (13). A recent review of 43 Ph+ ALL cases corroborates that *SETD2* mutations and *IKZF1* deletions are hallmark alterations in Ph+ ALL but unrelated to MPN biology (17), reinforcing the “two-hit” model of random co-occurrence. From a mechanistic perspective, the coexistence of ET and ALL may reflect a permissive BM microenvironment. Experimental studies suggest that MPN remodels the endosteal BM niche into a self-reinforcing leukemic niche through thrombopoietin, chemokine ligand 3 (CCL3), and direct cell-cell interactions (16). In the case reported here, NGS suggested mutations in the SETD2 gene and deletion of the IKZF1 gene, which are common molecular alterations in Ph+ ALL. Although baseline NGS was unavailable at ET diagnosis, no common MPN mutations (*SF3B1*, *U2AF1*, *TP53*, *IDH2*, and *EZH2*) were detected at Ph+ ALL transformation (18), further supporting clonal divergence. Future studies using single-cell sequencing could elucidate whether pre-ET hematopoietic stem cells harbor latent lymphoid-primed mutations.

In the pre-tyrosine kinase inhibitor (TKI) era, Ph+ ALL was typically resistant to conventional chemotherapy, with allo-HSCT being the only curative option. The long-term survival rate was only 10%–20% and even worse for elderly or unfit patients (19). The advent of TKIs and immunotherapies has revolutionized management. For frail patients, TKI-based regimens with reduced-intensity chemotherapy or corticosteroids achieve high remission rates. The EWALL-PH-01 study reported 96% complete remission (CR) and 36% 5-year overall survival (OS) with dasatinib plus low-dose chemotherapy in 71 patients with Ph+ ALL, with a median age of 69 years. Furthermore, 65% of patients achieved a 3-log reduction in *BCR::ABL1* transcript levels during consolidation (20). In 2020, Foà et al. used a dasatinib combined with a corticosteroid induction regimen followed by two cycles of blinatumomab to treat 63 patients with Ph+ ALL, achieving 18-month OS and disease-free survival (DFS) rates of 95% and 88%, respectively (21). Our patient had an atypical *BCR::ABL1* fusion gene (e13a3). Whether such patients respond similarly to TKIs and CD3/CD19 bispecific antibodies as those with common *BCR::ABL1* fusion genes is currently unreported. In chronic myeloid leukemia (CML) patients, the available literature suggests that e13a3/e14a3 transcripts correlate with superior imatinib responses (22, 23). The patient reported here experienced unstable angina upon admission. Acute leukemias, particularly with hyperleukocytosis, are associated with endothelial injury, hyperviscosity, and thromboinflammatory complications. These mechanisms may precipitate microvascular angina even in the absence of obstructive coronary artery disease (24, 25). Olverembatinib is a novel third-generation TKI approved in China (26). In a multi-center study, 20 patients with *de novo* Ph+ ALL were treated with olverembatinib-based regimens as frontline therapy. All patients achieved CR, and 85% achieved CMR within 3 months (27). Olverembatinib is chosen due to its superior safety profile and is prioritized over other TKIs in patients with cardiovascular comorbidities (26, 27). Our patient declined allo-HSCT, and blinatumomab was later introduced during consolidation to target residual disease, as supported by the EWALL-PH-01 trial (21). The patient achieved sustained molecular remission with olverembatinib and blinatumomab, avoiding intensive chemotherapy—a strategy aligned with emerging protocols for frail populations (21, 27). This contrasts with historical cases of ET-ALL treated with conventional regimens, where outcomes were poor (5, 10). While the patient currently refuses allo-HSCT, this remains a curative option for relapse, particularly with reduced-intensity conditioning (RIC) protocols (20). Additionally, emerging therapies such as CAR-T cells targeting CD19/CD22 may provide salvage options for refractory cases (28). In conclusion, this is a rare case of Ph+ ALL with e13a3 fusion transcripts following ET. It underscores the need for rigorous lineage evaluation in patients with ET with cytopenia or circulating blasts to exclude lymphoid transformation, which may mimic myelofibrosis progression. Molecular sequencing is important to elucidate whether pre-ET hematopoietic stem cells harbor latent lymphoid-primed mutations. Ph+ ALL with e13a3 transcripts may achieve durable remission with TKI-based regimens regardless of ET background. More data are needed to accumulate experience in treating Ph+ ALL following ET.

## Data Availability

The original contributions presented in the study are included in the article/supplementary material. Further inquiries can be directed to the corresponding author.
